# Cerenkov luminescence imaging is an effective preclinical tool for assessing colorectal cancer PD-L1 levels in vivo

**DOI:** 10.1186/s13550-020-00654-w

**Published:** 2020-06-15

**Authors:** Sheng Zhao, Wenbin Pan, Huijie Jiang, Rongjun Zhang, Hao Jiang, Zonghui Liang, Hongbo Hu

**Affiliations:** 1 Department of Radiology, The Second Affiliated Hospital of Harbin Medical University, Harbin, China; 2 Jiangsu Institute of Nuclear Medicine, Wuxi, China; 3 Jing’an District Centre Hospital of Shanghai, Fudan University, Shanghai, China

**Keywords:** Colorectal cancer, Immunotherapy, Cerenkov luminescence imaging, Programmed death ligand-1, Preclinical tool

## Abstract

**Background:**

Preclinical and clinical studies have demonstrated that immunotherapy has effectively delayed tumor progression, and the clinical outcomes of anti-PD-1/PD-L1 therapy were related to PD-L1 expression level in the tumors. A ^131^I-labeled anti-PD-L1 monoclonal antibody tracer, ^131^I-PD-L1-Mab, was developed to study the target ability of noninvasive Cerenkov luminescence imaging in colorectal cancer xenograft mice.

**Method:**

Anti-PD-L1 monoclonal antibody labeled with ^131^I (^131^I-PD-L1-Mab), and in vitro binding assays were used to evaluate the affinity of ^131^I-PD-L1-Mab to PD-L1 and their binding level to different colorectal cancer cells, and compared with flow cytometry, Western blot analysis, and immunofluorescence staining. The clinical application value of ^131^I-PD-L1-Mab was evaluated through biodistribution and Cerenkov luminescence imaging, and different tumor-bearing models expressing PD-L1 were evaluated.

**Results:**

^131^I-PD-L1-Mab showed high affinity to PD-L1, and the equilibrium dissociation constant was 1.069 × 10^-9^ M. The competitive inhibition assay further confirmed the specific binding ability of ^131^I-PD-L1-Mab. In four different tumor-bearing models with different PD-L1 expression, the biodistribution and Cerenkov luminescence imaging showed that the RKO tumors demonstrated the highest uptake of the tracer ^131^I-PD-L1-Mab, with a maximum uptake of 1.613 ± 0.738% IA/g at 48 h.

**Conclusions:**

There is a great potential for ^131^I-PD-L1-Mab noninvasive Cerenkov luminescence imaging to assess the status of tumor PD-L1 expression and select patients for anti-PD-L1 targeted therapy.

## Background

Anti-tumor immunity is a dynamic process of constant rebalancing. The anti-tumor immunity inhibits the tumor growth, while the tumor evades anti-tumor immunity by modifying the surrounding tumor microenvironment (TME) [1]. Antigen-presenting cells (APCs) activate T cells through two signals to eliminate heterologous antigens in the body. The first signal is between the antigen-loaded major histocompatibility complex (MHC) molecules and the T cell receptor (TCR) to identify the antigens. B7-1/B7-2 protein that is expressed on the surface of APCs bind to the co-stimulatory molecule CD28, further activating the T cells to form the secondary signal. To limit the possibility of excessive immune response that lead to tissue damage, T cells produce inhibitory molecules as a negative feedback mechanism, including the programmed cell death-1 (PD-1) [2]. The PD-1 protein has two ligands, programmed cell death ligand-1 (PD-L1) and programmed cell death ligand-2, in which PD-L1 expresses on the surface of tumor cells and bind to PD-1-expressing T cells, causing T cell exhaustion and evasion of immune surveillance. PD-L1 also binds to CD80, which competitively inhibits CD80-ligand-bound T-cell activation pathways [3].

Colorectal cancer (CRC) is one of the most common malignant neoplasms throughout the world, and its incidence and mortality ranked third among all the malignant neoplasms [4]. By 2030, its burden is expected to increase by 60%, and new cases were predicted to increase by 2.2 million, with 1.1 million cancer-related deaths [5]. Unfortunately, upregulated PD-L1 expression can lead to poor prognosis in CRC patients [6], but the immune checkpoint inhibitors (ICIs) therapy has opened a new window, brining hope to patients with high PD-L1 expression. Current clinical studies revealed that patients with MSI/dMMR mCRC and MSI/dMMR non-CRC chemoresistance metastasis demonstrated better efficacy with anti-PD-L1 therapy, with objective response rates of 40% and 57%, respectively [7]. More interestingly, PD-L1 is expressed in a variety of cancers, and preclinical studies on the use of immunotherapy for many malignant tumors have achieved better results. Due to exciting efficacy results in patients with advanced or unresectable melanoma, pembrolizumab has become the first FDA-approved ICI based on the PD-1/PD-L1 signaling pathway [8]. Till date, anti-PD-1/PD-L1 therapy has been confirmed as an effective strategy and approved for treating a vast number of malignant neoplasms, including triple negative breast cancer [9], small cell lung cancer [10], Hodgkin lymphoma [11], and cervical cancer [12]. The anti-PD-1/PD-L1 therapies demonstrate better response in patients, and this is related to tumor PD-L1 expression level in vivo [13]. For response stratification and ideal patient selection, it is necessary to detect the tumor PD-L1 expression level. It is worth noting that the tumor cells are surrounded by a homeostatic and dynamic TME that involves many uncertain factors including cytokine secretion, hypoxia, inflammation, and treatment responses, and changes in one of these factors affects tumor PD-L1 expression [14]. Hence, it is suspicious to determine PD-L1 expression on tissue samples by immunohistochemical (IHC) analysis, and a more effective PD-L1 detection method is urgently required. Molecular imaging with radiolabeled anti-PD-L1 antibodies comprehensively and dynamically assesses tumor PD-L1 expression in vivo, and monitors the possible changes in tumor PD-L1 expression during treatment.

Our prior study [15] used near-infrared dyes to optically label anti-PD-L1 monoclonal antibodies, confirming the feasibility of noninvasive monitoring of PD-L1 expression in vivo. Therefore, this study aimed to develop a ^131^I-labeled anti-PD-L1 monoclonal antibody to determine the possibility of noninvasive imaging to evaluate PD-L1 expression levels of CRC in vivo. In the future, imaging information obtained through this technology assists in selecting potential beneficiaries and predicting treatment responses for anti-PD-L1 therapy in patients with CRC.

## Methods

### Cell culture

All human CRC cells were kindly provided by Stem Cell Bank, Chinese Academy of Sciences (Shanghai, China) and maintained in a humidified incubator at 37 °C in 5% CO_2_. SW620 was cultured in RPMI 1640 medium (BI, CT, USA) supplemented with 10% fetal bovine serum (FBS; BI, CT, USA) and 1% Penicillin–Streptomycin (P/S). LS74T and LoVo cells were cultured in DMEM medium (Gibco, NY, USA) with 10% FBS and 1% P/S. RKO cells were maintained in MEM medium (Gibco, NY, USA) supplemented with 10% FBS, 1 mmol/L sodium pyruvate (Gibco, NY, USA) and 1% P/S.

### Western blot analysis

LoVo, SW620, LS174T, and RKO were lysed in radioimmunoprecipitation assay (RIPA) lysis buffer with 1 mmol/L PMSF on ice for 30 min. Equal amounts of total cellular protein (30 μg) were dissolved in SDS-PAGE gel and then transferred onto polyvinylidene fluoride (PVDF) membranes (Merck Millipore, Germany). The PVDF membranes were blocked with phosphate buffered saline with Tween® 20 (PBST) containing 5% BSA for 1 h followed by incubation overnight at 4 °C with anti-PD-L1 antibody (#ab205921, Abcam, Tokyo, Japan) at 1/200 dilution and anti-β-actin (Proteintech, Wuhan, China) at 1/5000 dilution. After washing with PBST, the membranes were incubated with goat anti-rabbit horseradish peroxidase (HRP)-conjugated secondary antibody (ZSGB-BO, Beijing, China) for 1 h at room temperature (RT). The bands were then detected by ChemiScope 6000 Exp chemiluminescence imaging system (Clinx, Shanghai, China), and β-actin quantitative normalization was performed using ImageJ 1.60 (NIH).

### Flow cytometry

Briefly, the cells were washed thrice with staining buffer (PBS containing 2 mmol/L EDTA and 0.5% FBS), and then the cells were incubated with recombinant anti-PD-L1 antibody (#ab205921, Abcam, Tokyo, Japan) or control IgG (#ab17273, Abcam, Tokyo, Japan) at 1/100 dilution for 30 min at RT after blocking with goat serum for 1 h. Next, the cells were washed and resuspended, stained with Alexa Fluor® 647-conjugated donkey anti-rabbit IgG secondary antibody (Biolegend, CA, USA) at 1/1000 dilution for 30 min at RT without light, and analyzed on a Cyto-FLEX flow cytometer (Beckman, CA, USA). At least 20,000 events were recorded and analyzed using FlowJo software VX0.7 (BD, NJ, USA).

### Immunofluorescence staining

The cells at a density of 5 × 10^5^ per cell line were cultured in 6-well plates with sterile glass slides until the cells were grown on the glass slides. The slides were then washed thrice with PBS and fixed in 4% paraformaldehyde for 15 min. Non-specific binding of antibodies was blocked by 10% goat serum for 30 min. The cells were incubated with anti-PD-L1 monoclonal antibody (#ab205921, Abcam, Tokyo, Japan) overnight at 4 °C. A secondary goat anti-rabbit antibody (Auragene, Changsha, China) conjugated with Dylight-488 was applied for 1 h at RT in a wet box. The nuclei were counterstained with DAPI medium. The images were obtained using an inverted fluorescence microscope (Nikon, Tokyo, Japan).

### Radiolabeling of antibody

For ^131^I radiolabeling, anti-PD-L1 monoclonal antibodies were used with Na^131^I (Chengdu Gaotong Isotope Co, China) and 1,3,4,6-tetrachloro-3α,6α-diphenylglycouril (Iodogen®, Sigma, MO, USA) as previously done in [16]. Briefly, in a tube coated with 50 μg Iodogen, 100 μL 6.91 nmol/L anti-PD-L1 monoclonal antibody (#HY-P9904, MCE, NJ, USA) 0.25 mol/L phosphate buffer (pH 7.6), and 18.5 MBq of Na^131^I were added into the tube. All components of the tube were blended frequently, and the reaction was carried out at RT for 15 min. Finally, the reaction system was added with 150 μL phosphate buffer and mixed well and left standing for 10 min to stop reaction. Subsequently, the contents were transferred onto a PD-10 column (GE Healthcare, MA, USA) and purified by using 20 mmol/L PBS containing 0.5% BSA as eluent (pH 7.4). A l-mL fraction of the eluate was collected, and the radioactivity in 1 μL aliquots of each fraction was measured by using a PerkinElmer 2480 automatic γ-counter, wherein the radiochemical purity was > 95%.

### In vitro assay

Human CRC cell lines LoVo, SW620, LS174T, and RKO were adjusted to a concentration of 5 × 10^6^/mL. RPMI-1640 containing 0.5% BSA was used as the binding buffer, followed by incubation of 100 μL fractions of cell suspension with 100 μL 47.5 pmol/L ^131^I-PD-L1-Mab (1.3 kBq) at 37 °C for 1 h as the test group (X group). The residual group (O group) and the total dose group (T group) for the controls were added with the same amount of ^131^I-PD-L1-Mab, and binding buffer was added to make the liquid volume equal. To determine the non-specific binding (NSB), ^131^I-PD-L1-Mab was incubated with RKO cells in the presence of a 2000-fold excess of unlabeled PD-L1 antibody. After cell incubation, the X group, O group, and NSB group were separated by centrifugation (1300 g, 10 min) to obtain the protein-binding fraction (pellet), and then the supernatants were removed. After centrifugation, the counts per minute (cpm) were measured in each group with a γ-counter (PerkinElmer). These were conducted in triplicate, and the cell-binding ratio was calculated using the formula

$$ \frac{X\mathrm{cpm}\left(\mathrm{NSBcpm}\right)-O\mathrm{cpm}}{T\mathrm{cpm}}\times 100\% $$

In a competitive inhibition assay, ^131^I-PD-L1-Mab (750 Bq) was incubated with RKO cells in the presence of varying concentrations of the unlabeled antibody (11.5–2300pmol/L) at 37 °C for 1 h within 1 mL of binding buffer. After incubation, the cell components were obtained by centrifugation, and the cell-associated activities were measured in a shielded well-type γ-counter. The IC50 value is defined as the concentration of unlabeled antibody that is required for 50% inhibition of radiolabeled antibody. In saturation binding assay, increasing concentrations of ^131^I-PD-L1-Mab (65–3333 Bq) were incubated with 5 × 10^5^ RKO cells in 1 mL binding buffer at 37 °C for 1 h. To detect non-specific binding, a 200-molar ratio of unlabeled antibody was used for co-incubation. Specific binding is defined as the binding of PD-L1 antibody and PD-1 antigen expressed on tumor cell membrane, which is equivalent to the difference between total binding and non-specific binding. The GraphPad Prism 7.00 software fits the curve on the relationship between specific binding and non-specific binding to determine PD-L1 receptor density of each cell and the dissociation constant (Kd) of the ^131^I-PD-L1-Mab.

### Animal model

Female BALB/C nu^-^/nu^-^ mice (6–8 weeks old) were obtained from Changzhou Cavens Laboratory Animal Co., Ltd. All animal protocols have been approved by the Animal Ethics Committee Board of Second Affiliated Hospital of Harbin Medical University (KY2018-215), and all procedures are under the National Institutes of Health guide for the care and use of Laboratory animals. Mice were housed in sterile cages with specific pathogen-free (SPF)-class animal facility on a 12-h light/dark cycle at 18 °C–23 °C in 50%–60% relative humidity. The mice had free access to food and drinking water and were transfected with 200 μL 2.5 × 10^7^/mL human CRC cells subcutaneously in their right flank. Tumors were grown for more than 21 days until the average tumor volume reaches to approximately 500 mm^3^.

### Ex vivo biodistribution

To determine the biodistribution and specificity of binding of ^131^I-PD-L1-Mab, two sets of study were performed in subcutaneous CRC cell line xenograft mice. Forty-eight hours before each study injection of ^131^I-PD-L1-Mab, 0.5% sodium iodide solution was used instead of drinking water to prevent the enrichment of ^131^I in mouse thyroids.

In the first study, subcutaneous LoVo, SW620, and LS174T xenograft mice were divided into three groups to receive intravenous injections of 7.5 kBq ^131^I-PD-L1-Mab, respectively. At 24 h, 48 h, and 120 h after ^131^I-PD-L1-Mab injection, the mice were euthanized, and 100 μL of blood was drawn from the carotid artery. The major tissues, organs, and tumor tissues of the mice were dissected and weighed, including the brain, heart, liver, spleen, lung, kidney, stomach, small intestine, colon, pancreas, muscle, adipose, bladder, and bone. All are placed in a γ-counting tube, and their radioactivity was measured using a γ-counter.

In the second study, subcutaneous RKO xenograft mice were divided into two groups. The control group was injected with 7.5 kBq ^131^I-PD-L1-Mab; in the blocking group, the subcutaneous RKO xenograft mice were injected with an excess of 300 μg of unlabeled PD-L1 antibody to block PD-L1 in vivo. The mice were euthanized at 48 h and 120 h after injection of ^131^I-PD-L1-Mab, and further processing was carried out according to the above steps. All ex vivo biodistribution results are expressed as percentage of injected activity per gram of tissue (%IA/g).

### Cerenkov luminescence imaging (CLI)

Before scanning Cerenkov luminescence imaging, 0.5% sodium iodide solution was used as described above. The mice that were inoculated with subcutaneous xenografts of LoVo, SW620, LS174T, and RKO cells received intravenous injections of 37 MBq ^131^I-PD-L1-Mab (protein dose 29.26 μg). At 24 h, 48 h, and 120 h after injection, the mice were anesthetized with 2% isoflurane. After that, the mice were subjected to CLI using the IVIS Spectrum Imaging System (PerkinElmer, MA, USA), and the parameters were set to Binning Factor 8, FOV 13.4 cm, exposure time 300 s. The mice were placed in supine position, with the tumors facing the lens during scanning and were continuously anesthetized. The images obtained from scanning were passed through the Living Image® 4.5 Software to determine the fluorescence intensity of tumors and background.

### Statistical analysis

All data were expressed as means ± SD, and statistical methods refer to “Guidelines for reporting of statistics for clinical research in urology” provided by Assel et al. [17].The difference in the uptake of ^131^I-PD-L1-Mab was assessed using ANOVA with Student–Newman–Keuls method multiple comparison test. The receiver operating characteristic (ROC) curves were drawn to evaluate the diagnostic efficacy of tumor uptake at different time points on tumors PD-L1 expression, and the areas under the curves (AUCs) at different time points were compared using the *U* tests. Statistical analysis was performed using GraphPad Prism version 7.00 and SPSS version 19.0 for Windows. A statistically significant difference was defined as *p* value of < 0.05.

## Results

### Different CRC cell lines have various levels of PD-L1 expression

**Fig. 1:**
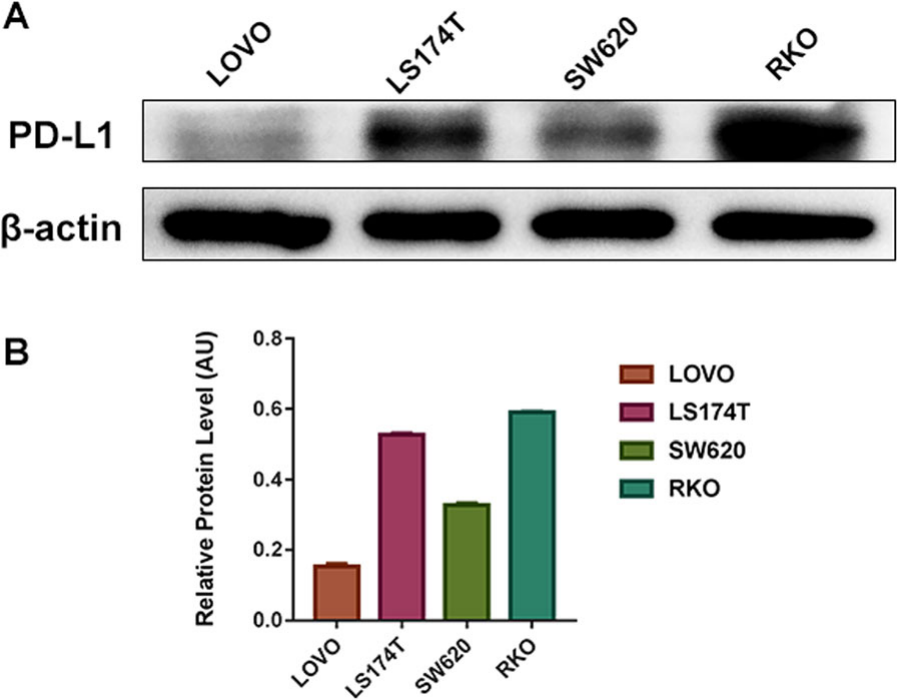
PD-L1 total protein expression analysis of four CRC cell lines. **a** Western blot analysis of total cell lysates using anti-PD-L1 and anti-β-actin antibody between LoVo, LS174T, SW620, and RKO in vitro. **b** The ratio of PD-L1 total protein intensity. Data are expressed as means ± SD, *** *p* < 0.001 (*n* = 3)

**Fig. 2:**
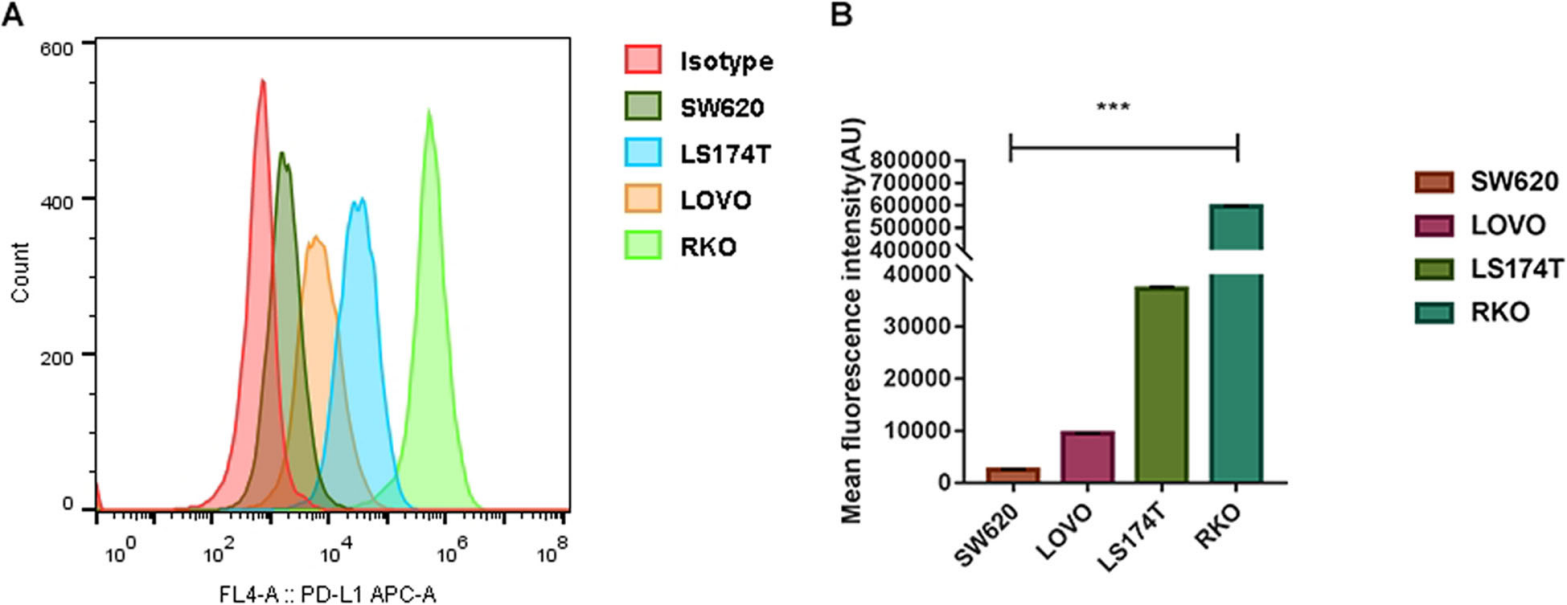
The differences in the PD-L1 expression on the plasma membrane among LoVo, LS174T, SW620, and RKO was evaluated by flow cytometry analysis. **a** The analysis of anti-human PD-L1 antibody binding to the PD-L1 of plasma membranes in LoVo, LS174T, SW620, and RKO cell lines. **b** Statistical summary of plasma membrane PD-L1 expression on four different CRC cell lines. Data are expressed as means ± SD, *** *p* < 0.001

**Fig. 3:**
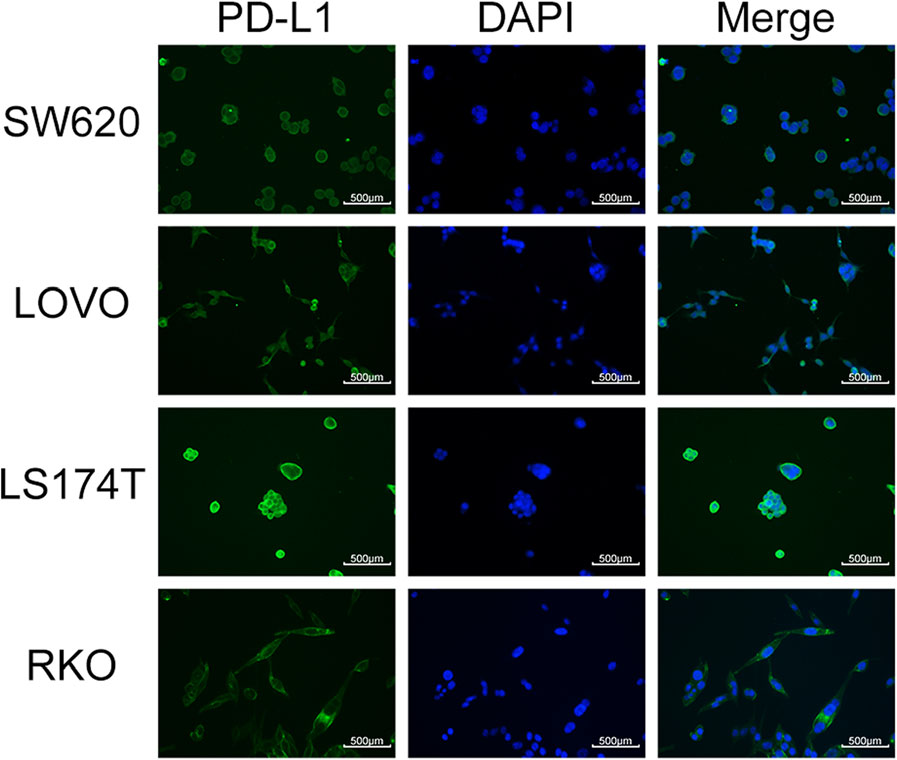
The subcellular localization of PD-L1 protein in LoVo, LS174T, SW620, and RKO was determined by immunofluorescence staining with anti-PD-L1 antibody (green) and DAPI nuclear staining (blue). PD-L1 protein is mainly located on the plasma membrane among the four cell lines, and a small amount of PD-L1 expression was observed in the cytoplasm

To determine the expression of PD-L1 protein in four human CRC cell lines (LoVo, LS174T, SW620, and RKO) in vitro, Western blotting, flow cytometry, and immunofluorescence staining were conducted. The Western blotting results showed (Fig. 1) various levels of endogenous PD-L1 expression among the four cell lines, in which the RKO cells (0.591 ± 0.006) showed the highest expression, followed by LS174T (0.527 ± 0.005), SW620 (0.329 ± 0.006), and LoVo (0.153 ± 0.009), and the difference was statistically significant (*p* < 0.001). To further detect the expression of PD-L1 on the plasma membrane among the four cell lines, the mean fluorescence intensity of the four cell lines (Fig. 2) was measured by flow cytometry and ranked as high to low: RKO, LS174T, SW620, and LoVo (595500 ± 2121.320, 372325.0 ± 374.059, 9533.0 ± 35.355, 2523.5 ± 67.175, respectively; *p* < 0.001). Also, immunofluorescence staining proved that PD-L1 protein was mainly located on the plasma membrane in the four cell lines, and a small amount of PD-L1 expression was observed in the cytoplasm (Fig. 3). In these four CRC cell lines, the diversity of PD-L1 protein expression was confirmed by Western blotting and flow cytometry results, and the graded expression was shown as RKO, LS174T, SW620, and LoVo, respectively.

### Specific binding characteristics of ^131^I-PD-L1-Mab and PD-L1 in vitro

**Fig. 4:**
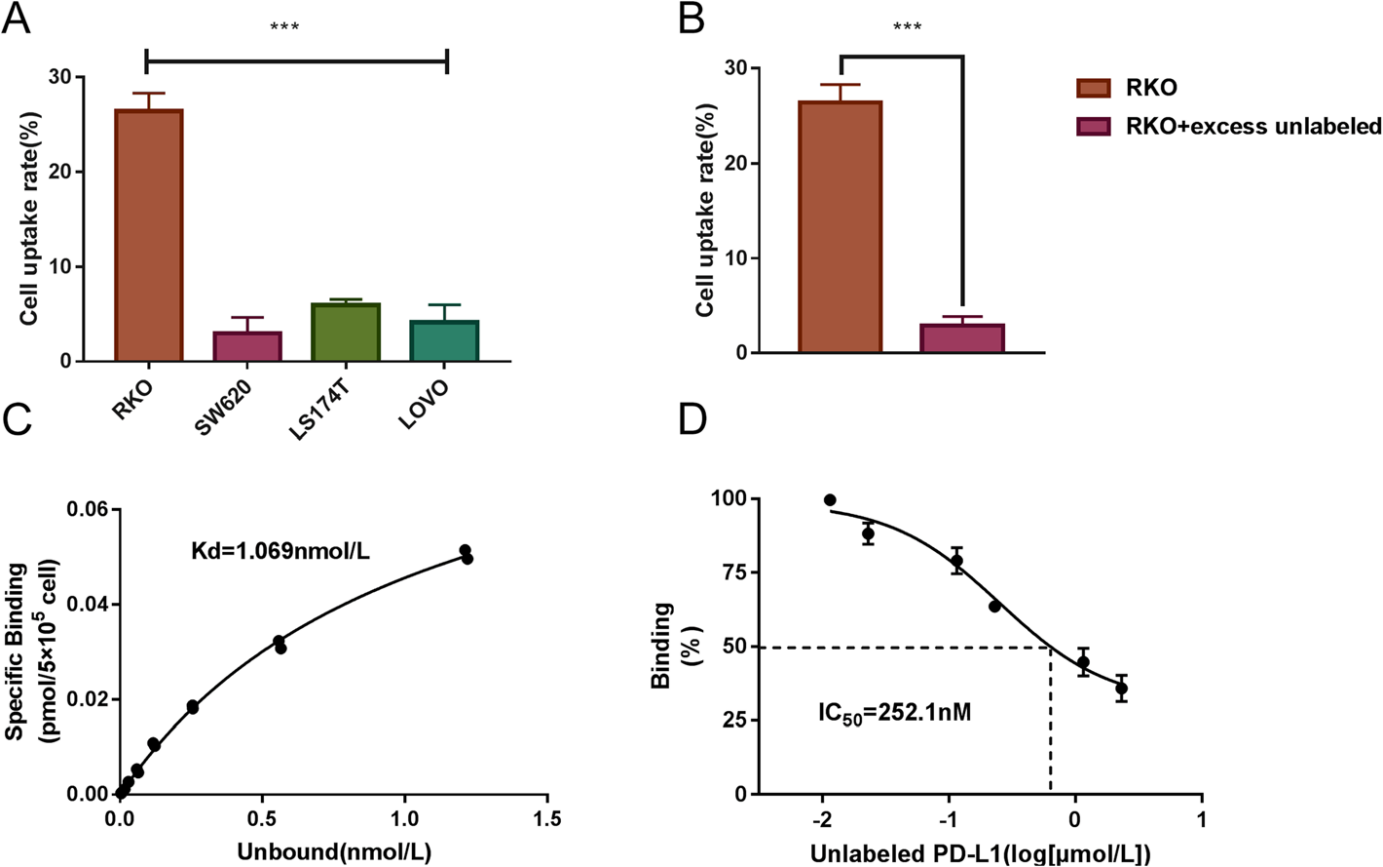
Cell affinity characteristics of ^131^I-PD-L1-Mab in vitro. **a** Cell binding of ^131^I-PD-L1-Mab to four different CRC cell lines with constant concentration of ^131^I-PD-L1-Mab. **b** Cell binding of ^131^I-PD-L1-Mab to RKO with or without blocking. **c** Saturation binding assay of ^131^I-PD-L1-Mab, Kd = 1.069 nmol/L, the number of binding sites was 113,671 ± 4183 per cell. **d** Competitive inhibition assay of ^131^I-PD-L1-Mab showed an IC50 of 252.1 nmol/L. The data are expressed as means ± SD, *** *p* < 0.001

Firstly, ^131^I-labeled PD-L1 antibody was used for cell binding assay along with a constant number of cells (5 × 10^5^) and a constant ^131^I-PD-L1-Mab concentration (47.5 pmol/L). As shown in Fig. 4a, the cell binding ratio of ^131^I-PD-L1-Mab to RKO, SW620, LS174T, and LoVo cells was 26.39%, 2.96%, 4.94%, and 4.14%, respectively (*p* < 0.001). Next, a 2000-fold excess of unlabeled PD-L1 antibody was added to ^131^I-PD-L1-Mab-incubated RKO cells, and the cell binding rate of ^131^I-PD-L1-Mab to RKO was decreased from 26.39% to 2.88% (Fig. 4b).

Secondly, based on the characteristics of RKO to determine the high PD-L1 expression in vitro and the high binding rate in the binding assay, RKO cells were selected for conducting ^131^I-PD-L1-Mab saturation binding assay and competitive inhibition assay. In saturation binding assay, using the method invented by Scatchard, the number of binding sites was quantitatively determined and was 113671 ± 4183 sites per cell, and the equilibrium dissociation constant of RKO was estimated to be 1.069 nmol/L. In competitive inhibition assay, the IC50 of the unlabeled PD-L1 antibody was 252.1 nmol/L. These data indicated that the ^131^I-PD-L1-Mab antibody specifically binds to tumor cells expressing PD-L1.

### Ex vivo biodistribution of ^131^I-PD-L1-Mab

**Fig. 5:**
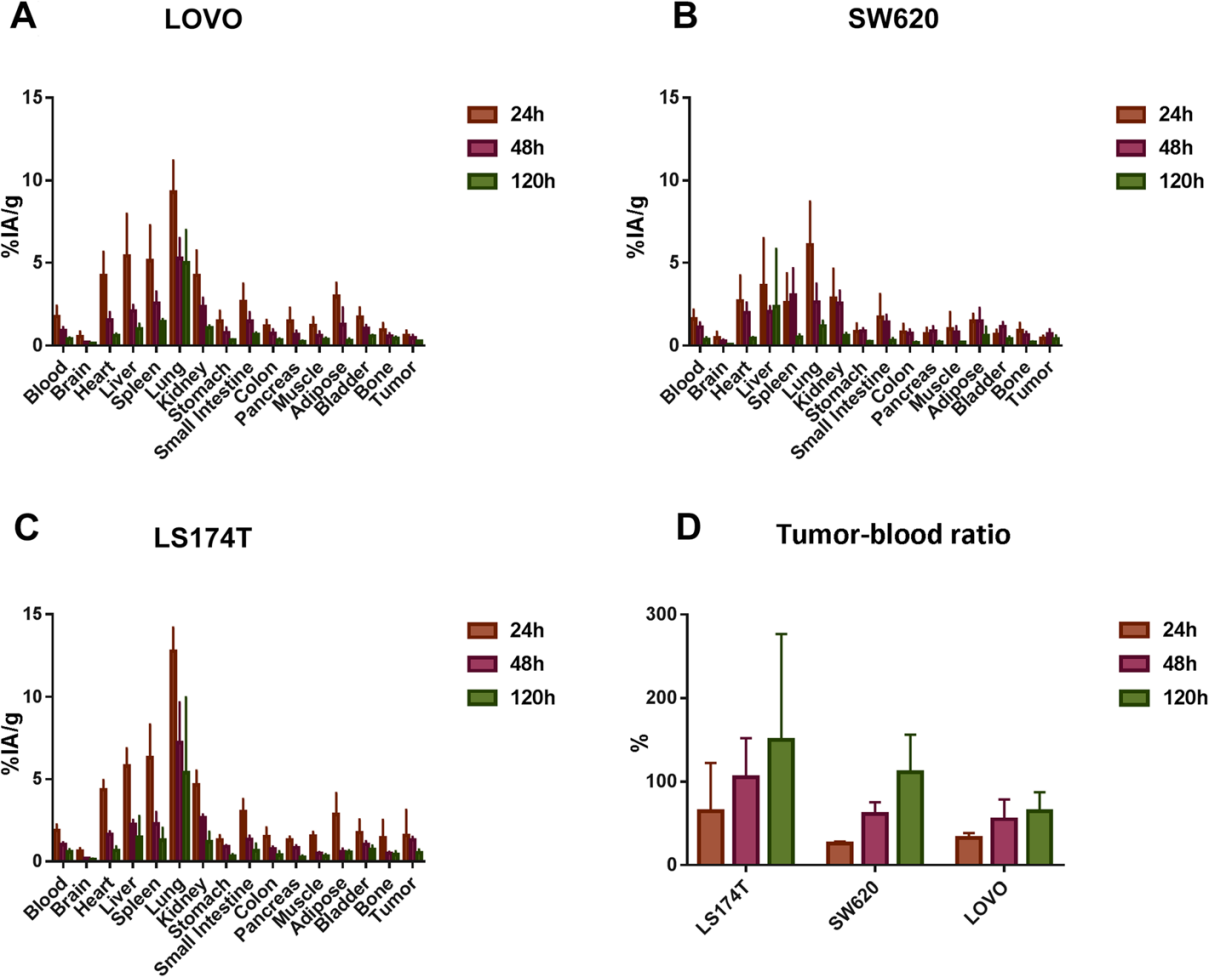
Ex vivo biodistribution of ^131^I-PD-L1-Mab in SW620 (**a**), LoVo (**b**), and LS174T (**c**) tumor-bearing nude mice at 24 h, 48 h, and 120 h after injection. The mean (%IA/g) ± SD at each time point after injection (*n* = 4 mice at each time point). **d** Corresponding quantitative data of the tumor (%IA/g)-to-blood (%IA/g) ratio of SW620, LoVo, and LS174T tumor-bearing nude mice

**Fig. 6:**
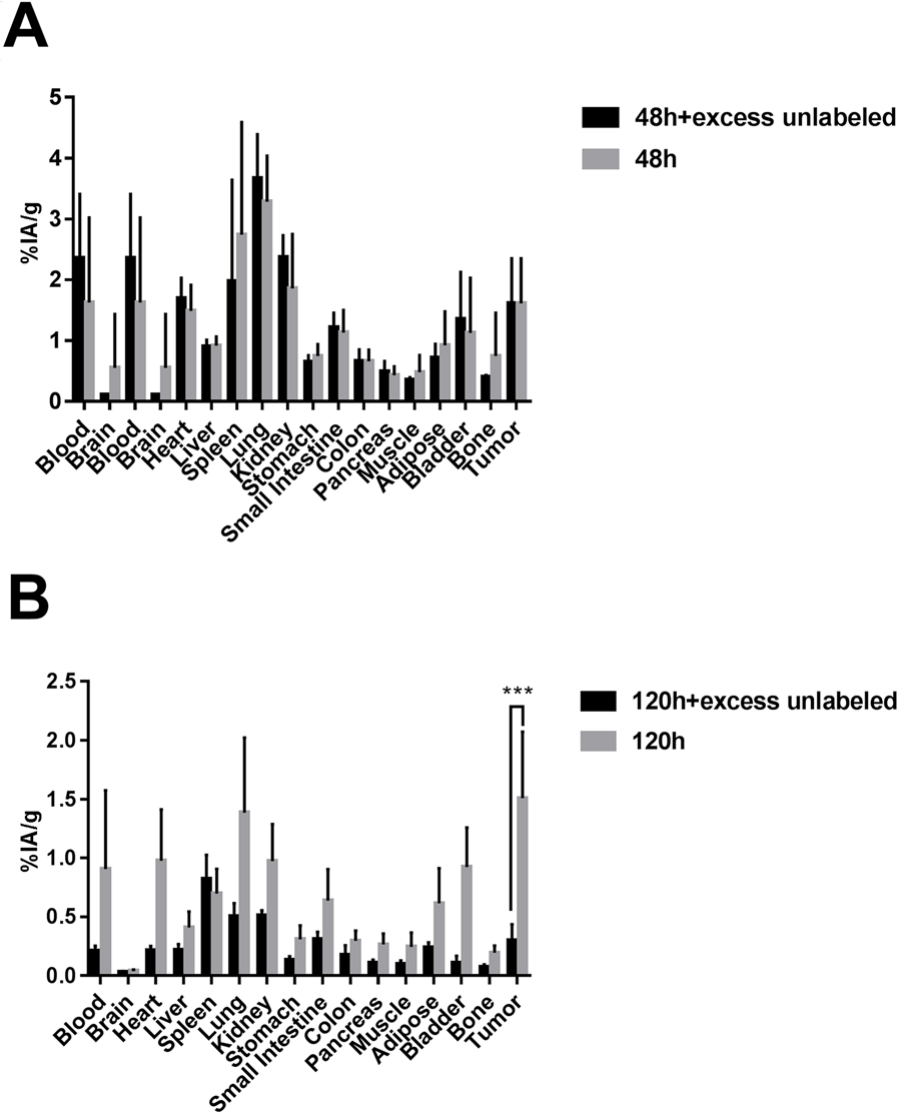
Comparison of ^131^I-PD-L1-Mab tumor uptake and post-blocking uptake in subcutaneous RKO xenograft mice. **a** After intravenous injection of ^131^I-PD-L1-Mab at 48 h, blocking group mice were injected with an excess of unlabeled PD-L1 antibody at the same time as ^131^I-PD-L1-Mab was injected. **b** After intravenous injection of ^131^I-PD-L1-Mab at 120 h, blocking group mice were injected with an excess of unlabeled PD-L1 antibody at the same time as ^131^I-PD-L1-Mab was injected. The data are expressed as means (%IA/g) ± SD at each time point after injection, *** *p* < 0.001

The biodistribution study was conducted using ^131^I-PD-L1-Mab in nude mice with SW620, LoVo, and LS174T xenograft tumors, the full tables of ex vivo biodistribution were available in the Supplement. At 24 h, the uptake of ^131^I-PD-L1-Mab in SW620 tumors was 0.427 ± 0.179%IA/g, which was increased to a maximum of 0.690 ± 0.299%IA/g at 48 h, but decreased to 0.411 ± 0.210%IA/g at 120 h. In contrast, in LoVo and LS174T tumors, the uptake was decreased over time, with 0.602 ± 0.322%IA/g and 1.580 ± 1.553%IA/g at 24 h, respectively (Fig. 5). The normal main organs with high uptake included lung, liver, and spleen, and the uptake were gradually decreased with time. Among them, the lung had the highest uptake, and the nude mice with LS174T xenograft tumors at 24 h as examples showed the highest uptake in lung (12.747 ± 1.429%IA/g) among other normal main organs, followed by spleen (6.292 ± 2.023%IA/g), liver (5.784 ± 1.079%IA/g), and kidney (4.636 ± 0.877%IA/g). At all three time points, ^131^I-PD-L1-Mab uptake was significantly higher in LS174T tumors than in SW620 and LoVo tumors. Interestingly, the tumor–blood ratio (T/B) of SW620, LoVo, and LS174T tumors was increased with time, reaching the highest value at 120 h (Fig. 5d). These data indicated that ^131^I-PD-L1-Mab discriminates between high and low expression of PD-L1 in CRC tumors. In the blocking biodistribution study of nude mice with RKO xenograft tumors (Fig. 6), compared to T/B of the blocking group receiving excess unlabeled PD-L1 antibody, the T/B of the control group (94.783 ± 46.567%) was not significantly higher than that of the blocking group (71.276 ± 12.122%) at 48 h; however, the T/B of the control group (221.728 ± 125.311%) was increased significantly than that of the blocking group (144.148 ± 59.783%) at 120 h. These results suggest specific binding of ^131^I-PD-L1-Mab to RKO tumors.

### Cerenkov PD-L1 specific imaging visualizes and distinguishes xenografts with low and high PD-L1 expression

**Fig. 7:**
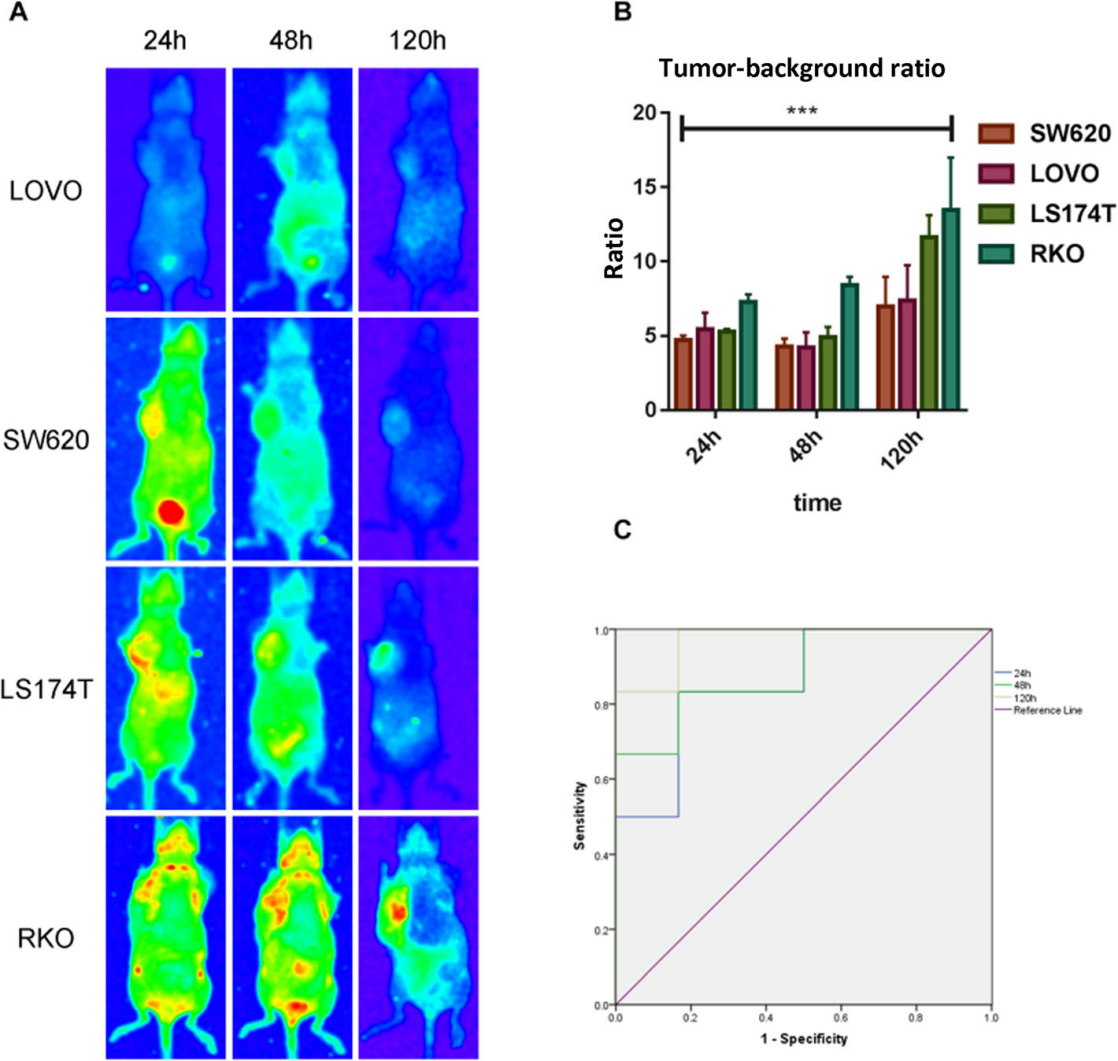
PD-L1-specific CLI at different time points in human CRC xenograft models with ^131^I-labeled PD-L1 antibody. **a** Cerenkov images of nude mice bearing four different CRC cell lines at 24 h, 48 h, and 120 h. **b** After injection of ^131^I-PD-L1-Mab (37 MBq), the areas of interest were delineated along the tumor margin at 24 h, 48 h, and 120 h to quantitatively measure the Cerenkov fluorescence intensity of the tumor-to-background ratio. **c** The diagnostic effectiveness of Cerenkov images was tested by receiver operating characteristic curves. The data are expressed as means ± SD, *** *p* < 0.001

^131^I-PD-L1-Mab PD-L1 specific imaging clearly visualized LoVo, LS174T, SW620, and RKO xenografts. The tumor–background ratio (TBR) was calculated as the average radiance ratio of equivalent ROIs on tumor and non-tumor background areas, and TBR increased significantly with increasing time (Fig. 7), reaching maximum at 120 h. Similarly, the implanted tumors better displayed at 120 h. The TBR remained the highest at 120 h after injection of ^131^I-PD-L1-Mab in RKO tumors (13.471 ± 3.571), followed by LS174T (11.630 ± 1.473), LoVo (7.403 ± 2.337), and SW620 (7.015 ± 1.975). The TBR increases over time, and this trend was consistent with the tumor-to-blood ratio in the ex vivo biodistribution study. Cerenkov imaging showed high-to-moderate uptake of ^131^I-PD-L1-Mab in RKO and LS174T tumors and low uptake in SW620 and LoVo tumors. This was confirmed in the ex vivo biodistribution study and was consistent with the Western blotting and flow cytometry results. These results demonstrated that RKO and LS174T tumors showed high PD-L1 expression, while SW620 tumors and LoVo tumors showed low PD-L1 expression. Based on the TBR of CLI obtained at 24 h, 48 h, and 120 h, the ROC analysis showed that the TBR can effectively discriminate the tumors with high and low PD-L1 expression. The AUCs were 0.861, 0.889, and 0.972, respectively, with no statistically significant difference in the AUCs at each time point (*p* > 0.05).

## Discussion

The traditional IHC analysis has many limitations in evaluating the tumor PD-L1 expression in patients. Also, the IHC scoring criteria are not uniform, and different manufacturers produce PD-L1 antibodies with different scoring criteria. There are four main clinical trial assays, including 28-8, 22C3, SP142, and SP263, in which the PD-L1 scores have lower consistency [18]. Secondly, during the process of tumor growth, metastasis, and apoptosis, the TME remains unstable, and PD-L1 expression showed spatiotemporal dynamics and furthermore showed intratumor and intertumor heterogeneity [19]. The IHC-based core needle biopsy only reflects PD-L1 expression level in a tiny fraction of a single tumor, but cannot comprehensively detect the situation of tumors throughout the body and the changes that might occur over time under conditions such as chemotherapy, radiotherapy, and immunotherapy. Finally, another important factor that interferes with immunotherapy is the disordered blood supply vessels surrounding the tumors. Although IHC analysis showed that some cancer patients are suitable to use ICIs (such as PD-L1 antibodies), effective drug concentration cannot be reached in their tumors internally, causing failed immunotherapy of tumors. In contrast, molecular imaging with radiolabeled anti-PD-L1 antibodies is considered to be more superior. Molecular imaging comprehensively detects PD-L1 expression in patients with tumors throughout the body, and analyzes the real-time dynamic analysis of possible changes in tumor PD-L1 expression during treatment. So far, many studies have used different radionuclides, such as (^99^mTc, ^68^Ga, ^64^Cu, ^89^Zr, and ^18^F) to label PD-L1 monoclonal antibodies [20, 21] or engineered PD-L1-specific peptides [22–24] in preclinical and clinical research. Some researchers have even found that atezolizumab labeled with ^89^Zr can predict the PFS and OS in patients better than the IHC-based core needle biopsy [21].

Recently, Cerenkov luminescence imaging is an emerging imaging strategy used to determine the location and margins of tumors during surgery [25] and has been used in radionuclide therapy monitoring [26, 27] and external beam radiotherapy plan making [28]. Compared with other optical imaging methods including our prior study [15], the fluorescence of CLI comes from a well-known physical phenomenon in the decay process of nuclides. It does not require external excitation light to irradiate and avoid the reduction of the signal-to-noise ratio caused by reflection of excitation light. More importantly, ^131^I reacts directly with the benzene ring hydroxyl adjacent to the antibody tyrosine residue, radiolabeling reaction slightly affected the molecular structure of antibody, ^131^I labeling method has passed the test of time and proved to be very simple and robust. On the other hand, the fluorescent dyes used in traditional optical imaging have large molecular weights and complex molecular structures, which may change the biological properties of the target molecules or even the biological behavior in vivo. Rijpkema et al. [29] labeled antibody with ^111^In and fluorescent dye using for a dual-modality imaging and found out when the near-infrared fluorophore to antibody ratio is over 3: 1, the tumor uptake is decreased and liver uptake is increased.

Since the PD-L1 antibody atezolizumab that is used for labeling is a full-molecule IgG, its molecular weight determines its biological half-life of several days. So ^131^I, a β- and γ-emitter with a long physical half-life (*T*_1/2_ = 8.021 days), has become our option for radiolabeling. The advantage of ^131^I is that it can be used as a clinical imaging agent and internal irradiation treatment drug for thyroid diseases and is widely used clinically and is relatively easy to obtain in the clinical and scientific research fields. To the best of our knowledge, our study is the first study to measure PD-L1 expression in human CRC subcutaneous xenograft mouse tumors using a ^131^I-labeled PD-L1 monoclonal antibody with CLI successfully [30]. By contrast, PET is a promising molecular imaging technique; it has a high spatial resolution and positioning capabilities; it can quantify the tumor uptake more accurately, but the disadvantage of this technique involves expensive equipment, long scanning time, and cumbersome preparation for positron nuclide. Similarly, SPECT can offer clinicians with a slice image representing the radioactive distribution in a layer of tissue, which is not affected by the depth, organ size, and thickness. Through computer reconstruction, it can provide three-dimensional images of patients’ entirety body. Meanwhile, like PET, SPECT has the ability to fuse with CT and further provide accurate corresponding anatomical and radioactive distribution images with CT fusion, not to mention the high sensitivity and fast imaging speed of SPECT. While CLI is cheap, simple to operate, and has higher throughput, the advantage of high throughput alone is of great significance in preclinical studies. For instance, typical CLI scans can image 5 animals at the same time in about 5 min. Conversely, PET scans of 1–2 animals often consumed 10–20 min; it represents CLI is quadrupled faster than small animal PET scanning [31]. Nevertheless, CLI has the inherent disadvantages of optical imaging. The Cherenkov radiation spectrum is mainly concentrated in the ultraviolet to blue region, which is more easily absorbed and scattered by biological tissues than PET and SPECT. Thus, this is why the spatial resolution, biological penetration, and signal specificity of CLI are worrisome. However, as a preclinical research method for small animal subcutaneous xenotransplantation, CLI still has high sensitivity and quantifiable analysis. Furthermore, this disadvantage can also be compensated by technological progress. Hu et al [32] developed Cherenkov luminescence tomography (CLT), which collects Cherenkov radiation in multiple directions and reconstructs images and can obtain the three-dimensional radioactive distribution in the organism. Then they implanted Na^131^I into mice as a radioactive source, by comparing the location of the implanted radioactive source reconstructed by SPECT and CLT; there was only a millimeter-level range in the later.

To develop a new Cerenkov luminescent tracer to detect PD-L1 expression in CRC, four different CRC cell binding assays were performed. The cell binding assay confirmed that RKO and LS174T demonstrated high to moderate expression levels of PD-L1, while SW620 and LoVo showed low levels of PD-L1expression, and the cell-binding ratio was consistent with western blotting analysis and flow cytometry. In the presence of excess unlabeled PD-L1 antibodies, the binding of ^131^I-PD-L1-Mab to PD-L1 on to the surface of tumor cells was significantly blocked. This showed that ^131^I-labeled monoclonal PD-L1 antibody still retained its immunoreactivity and can specifically bind to PD-L1. Secondly, a high affinity of ^131^I-PD-L1-Mab for PD-L1 was estimated by saturation binding assay (Kd = 1.069 nmol/L), and it is similar with that of the affinity of ^111^In-PD-L1.3.1 (Kd = 1.0 nmol/L) syntheses by Heskamp S, et al. [33]. However, it might be due to the source of antibody or different labeled nuclides that change the immune characteristics of the antibody, and the values of Kd are not identical. The immunoreactivity of ^131^I-PD-L1-Mab and its high affinity to PD-L1 laid foundation to its good characteristics for in vivo imaging.

Biological distribution analysis showed that RKO xenografts have high uptake of ^131^I-PD-L1-Mab; LS174T xenografts showed moderate uptake, while the uptake of LoVo and SW480 was decreased significantly, and the tumor and spleen uptake of RKO tumor-bearing mice were reduced by injecting excess unlabeled anti-PD-L1-Mab. This again verified that ^131^I-PD-L1-Mab specifically targets PD-L1 expression in tumor cells. Besides the tumor cells, PD-L1 also showed expression on hematopoietic cells [10] (such as B cells, T cells, megaphagocytes, and dendritic cells), in which the lung, spleen, and liver are rich. On the other hand, atezolizumab has cross-reactivity between human and mouse [34], and it is possible to explain as to why a large amount of ^131^I-PD-L1-Mab was observed in the lung, spleen, and liver. Similar results were also observed in studies using a mouse cross-reactive PD-L1 antibody such as atezolizumab [33–35] or using rat anti-mouse antibodies in a mouse tumor model [36]. The uptake of ^131^I-labeled monoclonal PD-L1 antibody in four xenograft models with different levels of PD-L1 expression differed from each other in vivo, and the Cerenkov fluorescence intensity of PD-L1 high expression in tumors was significantly higher than that of PD-L1 low expression, which in turn was affected by receiver operating characteristic curve analysis. The results also proved that PD-L1-specific CLI can effectively distinguish the level of PD-L1 expression in human CRC xenograft tumors. In blocking studies, we expect to observe a decrease in tumor uptake in the early stages to indicate specific binding to the target. However, we failed to observe similar results in the 48-h blocking study, which may be due to the sink of unlabeled PD-L1 antibody in the liver and spleen [37], failure to form a sufficient blood concentration to competitively inhibit uptake in the tumor, and tumor vascular disorder might further prevent antibodies to enter. Over time, labeled antibodies cleared and unlabeled antibodies competitively inhibited labeled antibodies on tumors gradually, thereby inhibiting tumor-labeled antibody uptake. This result can be reflected in the 120-h blocking study.

## Conclusion

In this study, a ^131^I-labeled anti-PD-L1 monoclonal antibody was synthesized to evaluate its properties both in vitro and in vivo.^131^I-PD-L1-Mab accumulates in tumors in high to moderate levels of PD-L1 expression and can be used in visualizing subcutaneously implanted xenografts. The CLI with ^131^I-PD-L1-Mab is not only considered as a prominent noninvasive imaging method that is accessible to assess and monitor dynamic PD-L1 expression in tumor lesions but is also used to clinically screen potential patients who can benefit from PD-1/ PD-L1 targeted therapy.

## Data Availability

The datasets used and/or analyzed during the current study are available from the corresponding author on reasonable request.

## Abbreviations

TME: Tumor microenvironment
APCs: Antigen-presenting cells
MHC: Major histocompatibility complex
TCR: T cell receptor
PD-1: Programmed cell death-1
PD-L1: Programmed cell death ligand-1
CRC: Colorectal cancer
ICIs: Immune checkpoint inhibitors
IHC: Immunohistochemical
NSB: Nonspecific binding
CLI: Cerenkov luminescence imaging
%IA/g: Percentage of injected activity per gram of tissue
T/B: Tumor–blood ratio
TBR: Tumor–background ratio
ROC: Receiver operating characteristic
AUCs: Areas under the curves
CLT: Cherenkov luminescence tomography

## References

[1] Kang TH, Mao CP, Kim YS, Kim TW, Yang A, Lam B (2019). TLR9 acts as a sensor for tumor-released DNA to modulate anti-tumor immunity after chemotherapy. J Immunother Cancer.

[2] Riella LV, Paterson AM, Sharpe AH, Chandraker A (2012). Role of the PD-1 pathway in the immune response. Am J Transplant.

[3] Butte MJ, Keir ME, Phamduy TB, Sharpe AH, Freeman GJ (2007). Programmed death-1 ligand 1 interacts specifically with the B7-1 costimulatory molecule to inhibit T cell responses. Immunity..

[4] Siegel RL, Miller KD, Jemal A (2019). Cancer statistics, 2019. CA Cancer J Clin.

[5] Arnold M, Sierra MS, Laversanne M, Soerjomataram I, Jemal A, Bray F (2017). Global patterns and trends in colorectal cancer incidence and mortality. Gut..

[6] Fakih M, Ouyang C, Wang C, Yiwey Tu T, Gozo MC, Cho M (2019). Immune overdrive signature in colorectal tumor subset predicts poor clinical outcome. J Clin Invest.

[7] Le DT, Uram JN, Wang H, Bartlett BR, Kemberling H, Eyring AD (2015). PD-1 Blockade in tumors with mismatch-repair deficiency. N Engl J Med.

[8] Robert C, Ribas A, Wolchok JD, Hodi FS, Hamid O, Kefford R (2014). Anti-programmed-death-receptor-1 treatment with pembrolizumab in ipilimumab-refractory advanced melanoma: a randomised dose-comparison cohort of a phase 1 trial. Lancet..

[9] Adams S, Diamond JR, Hamilton E, Pohlmann PR, Tolaney SM, Chang CW (2019). Atezolizumab plus nab-paclitaxel in the treatment of metastatic triple-negative breast cancer with 2-year survival follow-up: a phase 1b clinical trial. JAMA Oncol.

[10] Payandeh Z, Khalili S, Somi MH, Mard-Soltani M, Baghbanzadeh A, Hajiasgharzadeh K (2020). PD-1/PD-L1-dependent immune response in colorectal cancer. J Cell Physiol.

[11] Shen H, Yang ESH, Conry M, Fiveash J, Contreras C, Bonner JA (2019). Predictive biomarkers for immune checkpoint blockade and opportunities for combination therapies. Genes Dis.

[12] Chung HC, Schellens JHM, Delord J-P, Perets R, Italiano A, Shapira-Frommer R (2018). Pembrolizumab treatment of advanced cervical cancer: updated results from the phase 2 KEYNOTE-158 study. J Clin Oncol.

[13] Diggs LP, Hsueh EC (2017). Utility of PD-L1 immunohistochemistry assays for predicting PD-1/PD-L1 inhibitor response. Biomark Res.

[14] Locy H, de Mey S, de Mey W, De Ridder M, Thielemans K, Maenhout SK (2018). Immunomodulation of the tumor microenvironment: turn foe into friend. Front Immunol.

[15] Zhang M, Jiang H, Zhang R, Jiang H, Xu H, Pan W (2019). Near-infrared fluorescence-labeled anti-PD-L1-mAb for tumor imaging in human colorectal cancer xenografted mice. J Cell Biochem.

[16] Aherne GW, James SL, Marks V (1982). The radioiodination of bleomycin using iodogen. Clin Chim Acta.

[17] Assel M, Sjoberg D, Elders A, Wang X, Huo D, Botchway A (2019). Guidelines for reporting of statistics for clinical research in urology. BJU Int.

[18] Scheel AH, Dietel M, Heukamp LC, Jöhrens K, Kirchner T, Reu S (2016). Harmonized PD-L1 immunohistochemistry for pulmonary squamous-cell and adenocarcinomas. Mod Pathol.

[19] Eckel-Passow JE, Ho TH, Serie DJ, Cheville JC, Houston Thompson R, Costello BA (2020). Concordance of PD-1 and PD-L1 (B7-H1) in paired primary and metastatic clear cell renal cell carcinoma. Cancer Med.

[20] Christensen C, Kristensen LK, Alfsen MZ, Nielsen CH, Kjaer A (2020). Quantitative PET imaging of PD-L1 expression in xenograft and syngeneic tumour models using a site-specifically labelled PD-L1 antibody. Eur J Nucl Med Mol Imaging.

[21] Bensch F, van der Veen EL, Lub-de Hooge MN, Jorritsma-Smit A, Boellaard R, Kok IC (2018). 89 Zr-atezolizumab imaging as a non-invasive approach to assess clinical response to PD-L1 blockade in cancer. Nat Med.

[22] Kumar D, Pomper MG, Nimmagadda S (2019). Peptide-based PET quantifies target engagement of PD-L1 therapeutics - graphical abstract. The Journal of Clinical Investigation. J Clin Invest.

[23] Broos K, Keyaerts M, Lecocq Q, Renmans D, Nguyen T, Escors D (2017). Non-invasive assessment of murine PD-L1 levels in syngeneic tumor models by nuclear imaging with nanobody tracers. Oncotarget.

[24] Lv G, Sun X, Qiu L, Sun Y, Li K, Liu Q (2020). PET imaging of tumor PD-L1 expression with a highly specific nonblocking single-domain antibody. J Nucl Med.

[25] Spinelli AE, Schiariti MP, Grana CM, Ferrari M, Cremonesi M, Boschi F (2016). Cerenkov and radioluminescence imaging of brain tumor specimens during neurosurgery. J Biomed Opt.

[26] Geng C, Ai Y, Tang X, Shu D, Gong C, Guan F (2019). A Monte Carlo study of pinhole collimated Cerenkov luminescence imaging integrated with radionuclide treatment. Australas Phys Eng Sci Med.

[27] Spinelli AE, Ferdeghini M, Cavedon C, Zivelonghi E, Calandrino R, Fenzi A (2013). First human Cerenkography. J Biomed Opt.

[28] Jia MJ, Bruza P, Andreozzi JM, Jarvis LA, Gladstone DJ, Pogue BW (2019). Cherenkov-excited luminescence scanned imaging using scanned beam differencing and iterative deconvolution in dynamic plan radiation delivery in a human breast phantom geometry. Med Phys.

[29] Rijpkema M, Bos DL, Cornelissen AS, Franssen GM, Goldenberg DM, Oyen WJ (2015). Optimization of dual-labeled antibodies for targeted intraoperative imaging of tumors. Mol Imaging.

[30] Pang X, Liu M, Wang R, Liao X, Yan P, Zhang C (2018). Radioimmunoimaging and targeting treatment in an immunocompetent murine model of triple-negative breast cancer using radiolabeled anti–programmed death-ligand 1 monoclonal antibody. J Label Compd Radiopharm.

[31] Robertson R, Germanos MS, Li C, Mitchell GS, Cherry SR, Silva MD (2009). Optical imaging of Cerenkov light generation from positron-emitting radiotracers. Phys Med Biol.

[32] Hu Z, Liang J, Yang W, Fan W, Li C, Ma X (2010). Experimental Cerenkov luminescence tomography of the mouse model with SPECT imaging validation. Opt Express.

[33] Heskamp S, Hobo W, Molkenboer-Kuenen JDM, Olive D, Oyen WJG, Dolstra H (2015). Noninvasive imaging of tumor PD-L1 expression using radiolabeled anti-PD-L1 antibodies. Cancer Res.

[34] Chatterjee S, Lesniak WG, Gabrielson M, Lisok A, Wharram B, Sysa-Shah P (2016). A humanized antibody for imaging immune checkpoint ligand PD-L1 expression in tumors. Oncotarget.

[35] Truillet C, Oh HLJ, Yeo SP, Lee CY, Huynh LT, Wei J (2018). Imaging PD-L1 expression with immunoPET. Bioconjug Chem.

[36] Josefsson A, Nedrow JR, Park S, Banerjee SR, Rittenbach A, Jammes F (2016). Imaging, biodistribution, and dosimetry of radionuclide-labeled PD-L1 antibody in an immunocompetent mouse model of breast cancer. Cancer Res.

[37] Nedrow JR, Josefsson A, Park S, Ranka S, Roy S, Sgouros G (2017). Imaging of programmed cell death ligand 1: impact of protein concentration on distribution of anti-PD-L1 SPECT agents in an immunocompetent murine model of melanoma. J Nucl Med.

